# Phase 1b clinical trial of pucotenlimab (HX008), a novel anti-PD-1 monoclonal antibody, combined with gemcitabine and cisplatin in the first-line treatment of metastatic triple-negative breast cancer

**DOI:** 10.3389/fonc.2022.837963

**Published:** 2022-08-02

**Authors:** Jun Cao, Biyun Wang, Jian Zhang, Zhonghua Tao, Leiping Wang, Xichun Hu

**Affiliations:** ^1^ Department of Medical Oncology, Fudan University Shanghai Cancer Center, Shanghai, China; ^2^ Department of Oncology, Shanghai Medical College, Fudan University, Shanghai, China

**Keywords:** immunotherapy, pucotenlimab, gemcitabine, cisplatin, metastatic triple-negative breast cancer

## Abstract

**Background:**

Pucotenlimab, also called HX008, is a humanized anti-PD-1 antagonist IgG4 mAb. It blocks programmed cell death protein 1 (PD-1), programmed-death ligand 1 (PD-L1), and programmed death ligand-2 (PD-L2). In the CBCSG 006 trial, gemcitabine plus cisplatin (GP) has shown impressive antitumor activity as first-line therapy for metastatic triple-negative breast cancer (mTNBC). The phase 1b study was conducted to assess the safety and preliminary antitumor activity of pucotenlimab when combined with GP in patients with mTNBC in the first-line setting.

**Methods:**

Eligible patients with mTNBC with ≥6 months of DFI (disease-free interval) who have never received antitumor therapy for metastatic disease were screened. Participants received pucotenlimab at 3 mg/kg (d1, q3w) plus gemcitabine at 1,250 mg/m^2^ (d1, 8, q3w) and cisplatin at 75 mg/m^2^ (d1, q3w). Eligible patients received up to six cycles of pucotenlimab along with GP chemotherapy, while pucotenlimab could be maintained until disease progression or unacceptable toxicity occurred or withdrawal of informed consent. This study was registered in China under registration number CTR20191353.

**Results:**

Between July 2019 and March 2020, 31 patients were enrolled in this study. The median age was 50 (range 28–68) years. Among 31 patients who were evaluated, 25 (80.6%) experienced objective response and the other six (19.4%) experienced stable disease (SD). As of 4 August, the median progression-free survival (PFS) was 9.0 months (95% CI, 6.2–9.2). The most common grade 3 or 4 treatment-related adverse events included neutropenia (74.1%), anemia (35.5%), thrombocytopenia (32.3%), hypocalcemia (9.7%), hypokalemia (9.7%), and alanine aminotransferase increased (6.5%). There were no treatment-related deaths.

**Conclusion:**

Pucotenlimab plus GP demonstrated promising activity and a manageable safety profile in patients with mTNBC in the first-line setting.

## Introduction

Triple-negative breast cancer (TNBC) has a poor prognosis compared with other subtypes and accounts for 12–17% of all breast cancers (1). The median overall survival (OS) of patients with mTNBC is poor (8–13 months) (2, 3). It has been acknowledged to be a heterogeneous disease that exhibits significant differences, such as biological behavior, pathological characteristics, and gene expression profiles (4, 5). Several previous studies have investigated the molecular subtyping of TNBC and potential targets in different TNBC subgroups (6, 7). In Fudan University Shanghai Cancer Center (FUSCC), we also did comprehensive research on the genomic and transcriptomic landscape of Chinese TNBC, which is named as FUSCC subtype (8). Chemotherapy is recommended as the main systemic treatment in mTNBC, although with suboptimal efficacy and considerable toxicity. Novel therapy is urgently needed. Immunotherapy has been a popular and promising treatment strategy for a variety of tumors, such as lung cancer, cervical cancer, urothelial carcinoma, and so on. It was reported that PD-L1 and tumor-infiltrating lymphocytes (TILs) showed prognostic value in early TNBC (9–11). A higher percentage of TILs was also supposed to be associated with response to atezolizumab in earlier lines of treatment, particularly in the first-line in mTNBC (12). However, PD- L1/PD-1 antibodies were demonstrated with low response rates when used as single-agent treatment in previous studies (13–15). Chemotherapy can induce tumor antigen release. As a result, it may enhance the antigenicity of cancer cells, upregulate PD-L1 expression level on tumor cells, and increase the number of CD8+ TILs (16, 17). According to the results of the Impassion 130 study (18) and the Keynote 355 study (19), both atezolizumab and pembrolizumab showed promising efficacy for patients with PD-L1 positive mTNBC in the first-line setting. Gemcitabine plus cisplatin (GP) has shown satisfying antitumor activity in CBCSG 006 trial (ClinicalTrials.gov, NCT01287624) (20) and has been recommended as a standard treatment in the first-line setting for mTNBC in various Chinese breast cancer guidelines and German Gynecological Oncology Group (AGO) recommendations (21).

Pucotenlimab is a humanized IgG4 anti-PD-1 monoclonal antibody with an engineered Fc domain that blocksPD-1, PD-L1, and PD-L2 (22). As it uses the IgG4 Fc isotype, it has no antibody-dependent cell-mediated cytotoxicity (ADCC) and complement-dependent cytotoxicity (CDC) activity, which can avoid killing PD-1-expressing immune cells. Pucotenlimab showed significant inhibition of tumor growth both *in vivo* and *in vitro*, which suggests that it is a promising candidate for immunotherapy (22, 23). Based on the pharmacokinetics from a phase I trial, pucotenlimab had an acceptable toxicity profile and 3 mg/kg or 200 mg every 3 weeks is recommended as an optimal dose for further drug development (24).

The phase 1b study was conducted to assess the safety and preliminary antitumor activity of pucotenlimab, along with GP, in Chinese patients with mTNBC in a first-line setting.

## Materials and methods

### Eligibility criteria

Eligible patients were ≥18 and ≤70 years of age with histologically confirmed TNBC either from the primary or metastatic tumor, with stage IV disease, and with ≥6 months of DFI and naive to previous systemic treatment for metastatic disease. Triple-negative was defined as <1% staining in the nuclei of ER and PR expression and no overexpression of HER2, which were all judged according to American Society of Clinical Oncology–College of American Pathologists guideline criteria (25, 26). The establishment of ER, PR, and HER2 status was done preferentially with tissue from recurrent or metastatic lesions, but the primary breast cancer tissue was also acceptable when the metastatic biopsy specimen was unavailable for irresistible reasons. Additional key eligibility criteria include Eastern Cooperative Oncology Group performance status (ECOG PS) of 0 or 1; at least one measurable lesion assessed by Response Evaluation Criteria in Advanced Solid Tumors version 1.1 (RECIST v1.1) at baseline; adequate organ function and a life expectancy of 3 months or more. Previous adjuvant or neoadjuvant settings were acceptable, but they should be completed at least 6 months before metastatic disease is diagnosed. The main exclusion criteria included symptomatic or unstable CNS metastases; active or history of autoimmune disease; history or active interstitial lung disease or pulmonary fibrosis; prior treatment with anti-PD-1/PD-L1, anti-CTLA-4, or other similar agents; and adverse events (AEs) from previous therapy that had not recovered to grade 1 or less according to the National Cancer Institute Common Terminology Criteria for Adverse Events (NCI CTCAE, version 5.0).

### Study design and treatment

This study was an open-label, single-arm, phase Ib study of pucotenlimab combined with gemcitabine and cisplatin as first-line treatment for patients with mTNBC. Eligible patients received pucotenlimab 3 mg/kg by intravenous infusion on day 1, gemcitabine at 1,250 mg/m^2^ on days 1 and 8, and cisplatin 75 mg/m^2^ on day 1. A maximum of six cycles of GP chemotherapy was given, while HX008 could be maintained until disease progression, unacceptable toxicity occurred, or withdrawn according to the decisions of patients.

The study protocol was approved by the Ethics Committee of FUSCC. The study was conducted in accordance with the Declaration of Helsinki. All patients provided written informed consent before enrollment. The trial is registered in China under registration number CTR 20191353.

### Endpoints

The primary endpoint was safety. The secondary endpoints were progression-free survival (PFS, defined as the time from the date of randomization to progression or death from any cause, whichever occurred first), objective response rate (ORR, defined as a best response of complete or partial response from the start of treatment until disease progression or death, in accordance with the RECIST 1.1 criteria), 6-month PFS rate, overall survival (OS, defined as the time from randomization to death from any cause), and pharmacokinetics parameters (not addressed in this article).

All AEs were recorded and followed up from the initiation of treatment to 30 days after the last dose of protocol treatment or the start date of subsequent anti-tumor therapy. AEs were graded according to NCI CTCAE, version 5.0.

Tumor response was assessed by investigators with CT or MRI, using Response Evaluation Criteria in Solid Tumors (RECIST Version 1.1) every 6 weeks until week 24, then every 8 weeks until discontinuation or up to 2 years, and then every 12 weeks for 1 year and every 24 weeks for subsequent years. Investigators evaluated tumor response according to immune-related RECIST (iRECIST) to determine whether subsequent treatment should be performed.

### Statistical analysis

This phase 1b study was designed to enroll at least 15 evaluable patients. More subjects would be enrolled if there were patients with treatment discontinuation for reasons other than safety. The full analysis set (FAS) is comprised of patients who successfully received at least one dose of pucotenlimab. Safety and efficacy will be statistically analyzed based on the FAS. Safety was analyzed using descriptive statistics. The median PFS and DOR (duration of response) were estimated using the Kaplan–Meier method. Data analyses were conducted using SAS statistical software (version 9.4).

## Results

### Baseline patient characteristics

From July 2019 to March 2020, 31 patients were enrolled in this study. All patients were evaluable and had received ≥1 dose of pucotenlimab combined with gemcitabine plus cisplatin. Baseline characteristics are listed in **Table 1**. The median age was 50 (range 28–68) years. Overall, 24 patients were histologically confirmed as triple-negative in the primary tumor tissue and the remaining seven patients in the metastatic tissue. Approximately 81% had visceral metastasis. Most patients had received adjuvant or neoadjuvant chemotherapy and had a disease-free interval (DFI, defined as the interval from the completion of primary breast cancer surgery to the diagnosis of recurrence or metastasis) of ≥1year.

**Table 1:** Baseline patient characteristics.

Age (years)	50 (28–68)
<40	8 (26%)
≥40	23 (74%)
**ECOG performance status**	
0	1 (3%)
1	30 (97%)
**Time between breast surgery and recurrent disease**	
Primary metastatic	2 (6%)
<1 year	4 (13%)
≥1 year	25 (81%)
**Number of metastatic organ sites**	
1	5 (16%)
2	9 (29%)
≥3	17 (55%)
Metastatic sites	
Lymph nodes	23 (74%)
Lung	20 (65%)
Bone	11 (35%)
Liver	11 (35%)
Chest wall or skin	6 (19%)
Contralateral breast	3 (10%)
Spleen	1 (3%)
Renal	1 (3%)
Adrenal	1 (3%)
Brain	1 (3%)
Visceral disease	25 (81%)
**Neoadjuvant or adjuvant chemotherapy**	
Anthracycline	27 (87%)
Taxane	26 (84%)
Anthracycline and taxane	25 (81%)

ECOG, Eastern Cooperative Oncology Group; ER, estrogen receptor; PR, progesterone receptor.

At the data cutoff (4 August 2021), the median follow-up duration was 18.1 months (range 4.2–21.8), with a median duration of treatment of 8.2 months (range 0.03–20.93). Patients received a median of 12 (range 1–31) cycles of pucotenlimab treatment and a median of 6 (range 2–6) cycles of chemotherapy. A total of 27 patients (87.1%) discontinued study treatment. The main reasons for discontinuation were disease progression (19/27), the decision of investigators (4/27), and the choice of patients (4/27). Four patients (12.9%) were still under treatment.

### Safety

All patients (31/31) were reported to have treatment-related adverse events. The most common treatment-related AEs (TRAEs) were leukopenia (100.0%), anemia (100.0%), neutropenia (96.8%), hypomagnesemia (93.5%), fatigue (93.5%), and anorexia (90.3%) (**Table 2**). Grade ≥3 TRAEs occurred in 23 patients (74.2%). The most frequent grade ≥3 TRAEs were neutropenia (74.1%), anemia (35.5%), thrombocytopenia (32.3%), hypocalcemia (9.7%), hypokalemia (9.7%), and alanine aminotransferase increased (6.5%). Serious adverse events (SAEs) included anemia (6.5%), thrombocytopenia (6.5%), alanine aminotransferase increased (6.5%), neutropenia (3.2%), anorexia (3.2%), fatigue (3.2%), hypokalemia (3.2%), and hypocalcemia (3.2%), occurred in seven (22.6%) patients, and all recovered after best supportive care. Immune-related TRAEs included hypothyroidism (32.3%), skin reactions (25.8%), hyperthyroidism (16.1%), and adrenal insufficiency (3.2%), nearly all of which were grade 1 or 2. Immune-related TRAEs are listed in **Table 3**.

**Table 2:** TRAEs of any grade occurring in ≥10% of patients.

	Any grade (%)	Grade ≥3 (%)
Any TRAE*	31 (100.0)	23 (74.2)
Treatment-related SAEs	7 (22.6)	4 (12.9)
TRAEs leading to discontinuation	1 (3.2)	1 (3.2)
TRAE leading to dose delay or reduction	21 (67.7)	12 (38.7)
Leukopenia	31 (100.0)	16 (51.6)
Anemia	31 (100.0)	11 (35.5)
Neutropenia	30 (96.8)	23 (74.2)
Hypomagnesemia	29 (93.5)	1 (3.2)
Fatigue	29 (93.5)	0
Anorexia	28 (90.3)	0
Hypertriglyceridemia	27 (87.1)	1 (3.2)
Thrombocytopenia	25 (80.7)	10 (32.3)
Alanine Aminotransferase increased	24 (77.4)	2 (6.5)
Hyponatremia	23 (74.2)	0
Nausea	22 (71.0)	0
Aspartate Aminotransferase increased	22 (71.0)	1 (3.2)
Hypocalcemia	20 (64.5)	3 (9.7)
Constipation	19 (61.3)	0
Hypercholesterolemia	19 (61.3)	0
Hyperglycemia	18 (58.1)	0
Alopecia	18 (58.1)	0
Hyperuricemia	18 (58.1)	0
Hypokalemia	17 (54.8)	3 (9.7)
Hypophosphatemia	17 (54.8)	0
Proteinuria	14 (45.2)	0
Weight loss	14 (45.2)	0
Vomiting	12 (38.7)	0
Hypercalcemia	11 (35.5)	0
Creatinine increased	9 (29.0)	0
Serum amylase increased	9 (29.0)	0
Pain	8 (25.8)	1 (3.2)
Fever	7 (22.6)	0
Insomnia	7 (22.6)	0

*TRAE, Treatment-related AEs.

All adverse events that occurred in at least 20% of patients are listed.

**Table 3:** Immune-related TRAEs.

	Any grade (%)	Grade ≥3 (%)
Hypothyroidism	10 (32.3)	0
Skin Reactions	8 (25.8)	1 (3.2)
Hyperthyroidism	5 (16.1)	0
Adrenal insufficiency	1 (3.2)	0
Total	7 (22.6)	1 (3.2)

Only one patient in this study discontinued the treatment because of TRAEs. Approximately 67.7% of patients had TRAEs, leading to delayed use of chemotherapy and/or pucotenlimab. The most common TRAEs causing delay were neutropenia (74.1%), thrombocytopenia (51.6%), anemia (29.3%), hypocalcemia (19.4%), alanine aminotransferase increased (9.7%), hypokalemia (9.7%), and fatigue (9.7%). Twelve patients received a dose reduction of chemotherapy for grade 3/4 thrombocytopenia (4/12), creatinine clearance decreased (3/12), anemia (2/12), alanine aminotransferase increased (2/12), and febrile neutropenia (1/12), which were all mainly considered related to chemotherapy. No patient experienced treatment-related fatal AEs.

### Efficacy

**Figure 1:**
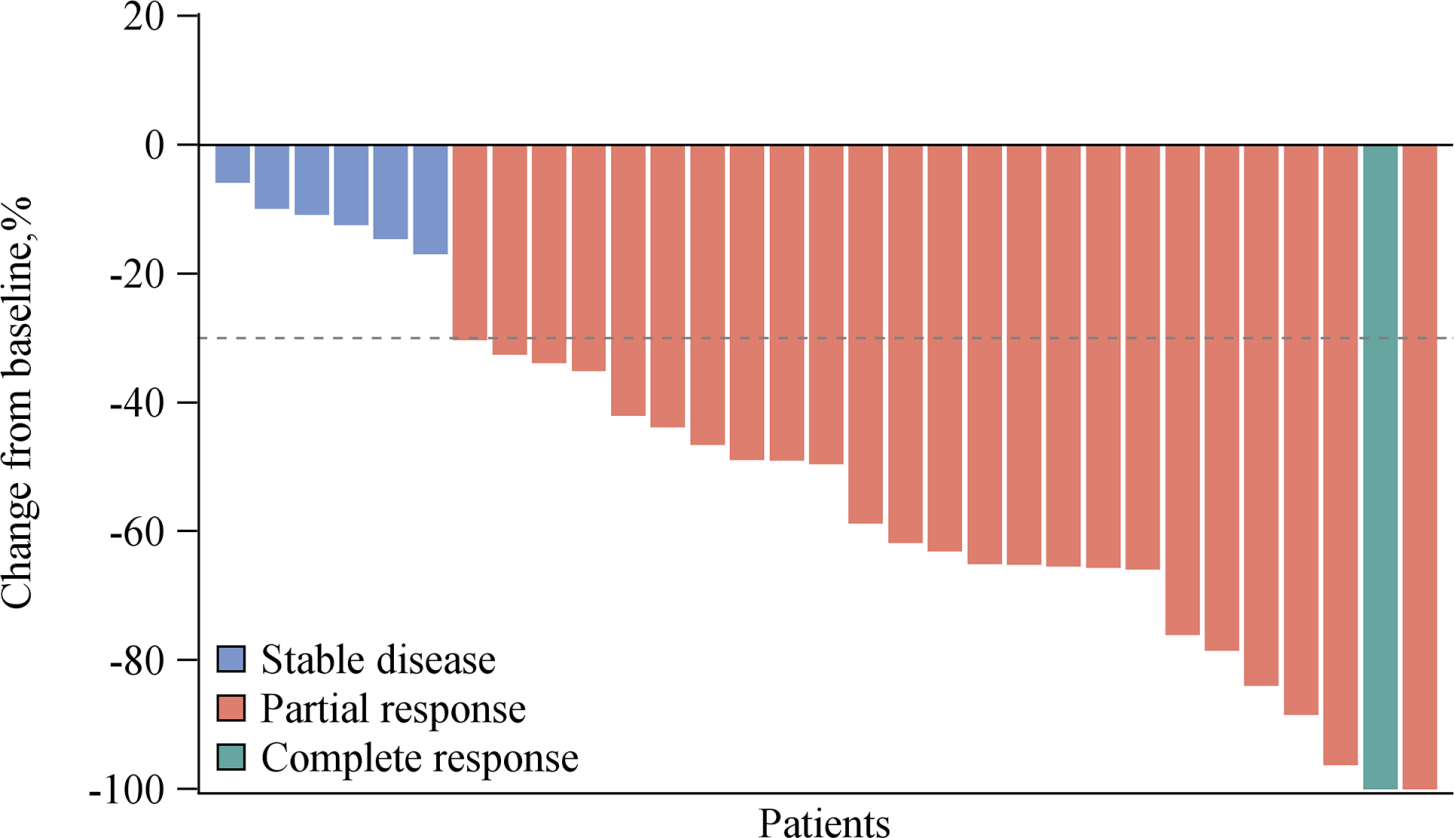
Overall response of pucotenlimab with gemcitabine plus cisplatin as assessed by investigators (N = 31).

**Figure 2:**
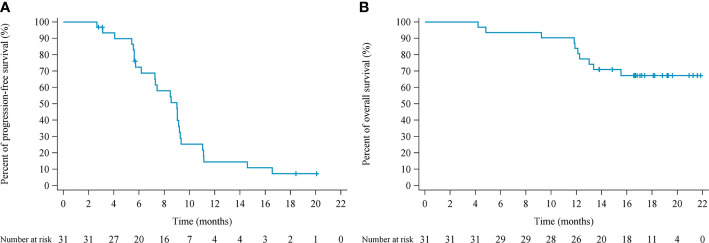
Kaplan–Meier estimates of progression-free survival **(A)** and overall survival **(B)**.

The efficacy of all 31 patients was evaluable. Among these, one achieved a complete response (CR), 24 achieved partial response (PR), and six achieved stable disease (SD) as their best responses, respectively (**Figure 1**), with ORR reaching 80.6% and DCR 100%. At the data cutoff, three patients were still receiving pucotenlimab treatment for continuing responses. The median DOR was 7.0 months (range 1.5–18.8). The median PFS was 9.0 months (95% CI 6.2–9.2) (**Figure 2**), and the 6-month PFS rate was 72.4% (95% CI 52.29%–85.16%). The median OS was not reached (95% CI 15.5, NE) after a median follow-up of 18.1 months (**Table 4**).

**Table 4:** Response and survival data (FAS population).

HX008+GP	
**response** n (%)	
Complete response	1 (3.2%)
Partial response	24 (77.4%)
Stable disease	6 (19.4%)
Progressive disease	0
Missing data or not assessable	0
Overall response	80.6% (62.53%–92.55%)
**Progression-free survival**	
Number of events	26
Median progression-free survival, months(95% CI)	9.00 (6.18–9.20)
**Overall survival**	
Number of events	10
Median overall survival, months (95% CI)	NE (15.54, NE)

## Discussion

This open-label, single-arm, phase Ib study of pucotenlimab combined with gemcitabine and cisplatin demonstrated a manageable safety profile and promising antitumor efficacy as first-line treatment in Chinese patients with mTNBC. The addition of pucotenlimab was well tolerated and did not increase the incidence and severity of TRAEs, which were generally consistent with those of the GP regimen (20). Our study extended observations with another novel PD-1 antibody, pucotenlimab, combined with a standard first-line chemotherapy regimen widely used in Chinese mTNBC daily practice, thereby providing additional treatment options to this population. Most AEs were grade 1/2. Hematotoxicity, including neutropenia, anemia, and thrombocytopenia, was the most frequently reported, which is supposed to be associated with gemcitabine and/or cisplatin. However, the incidences of any grade of serum electrolyte disturbance, such as hypomagnesemia, hyponatremia, hypocalcemia, hypokalemia, and hypophosphatemia, were higher than those in the CBCSG 006 study. Furthermore, hepatotoxicity, such as alanine aminotransferase increased and/or aspartate aminotransferase increased, was also reported more frequently in this study, which was supposed to be enhanced by pucotenlimab, although almost all of these increased TRAEs were grade 1/2. Immune-related toxicities, including hypothyroidism, skin reactions and hyperthyroidism, were comparable to studies with other anti-PD-1 antibodies with or without chemotherapy in breast cancer (14, 15, 18, 19). Only one patient in this study discontinued the treatment because of TRAEs. Approximately 67.7% of patients had TRAEs leading to delayed use of chemotherapy and pucotenlimab, mainly because of hematotoxicity, which was considered more related to chemotherapy. Twelve patients received a dose reduction of chemotherapy, mainly for thrombocytopenia, creatinine clearance decrease, and anemia, which were all considered related to chemotherapy.

The median PFS in this study was 9.0 months (95% CI 6.2–9.2), regardless of the expression status of PD-L1. Meanwhile, in the KEYNOTE 355 study (19), pembrolizumab combined with chemotherapy significantly improved PFS compared with chemotherapy alone in patients with combined positive score (CPS) ≥10 (9.7 months *vs*. 5.6 months, p = 0·0012). Chemotherapy allowed in this study included gemcitabine plus carboplatin (GC), single-agent of nab-paclitaxel or paclitaxel, according to the choice of the investigator. The subgroup analysis demonstrated that for patients with CPS ≥10, the PFS was all favored for the pembrolizumab-chemotherapy group regardless of the chemotherapy regimen used, although the longest PFS was 9.9 months from the pembrolizumab-nab-paclitaxel subgroup. In the Impassion 130 study (18), the median PFS for PD-L1-positive patients was 7.5 months with atezolizumab combined with nab-paclitaxel, compared with 5.0 months with nab-paclitaxel alone (p <0.001). It was reported that the proportion of mTNBC with CPS ≥10 was about 38% in the KEYNOTE 355 study (19) and PD-L1 expression on tumor-infiltrating immune cells was ≥1% [VENTANA PD-L1 SP142 immunohistochemical testing (27)] was about 41% in the IMPASSION 130 study (18), which still meant that only a small population would benefit from PD-1 or PD-L1 antibodies. Although there was no control group in this study, it suggested that the pucotenlimab plus GP regimen demonstrated potential efficacy in the first-line therapy in the whole mTNBC population, when compared with response and survival data from other classical trials held in similar populations listed in **Table 5**.

**Table 5:** Response and survival data from some classical phase III trials in the first-line setting in mTNBC.

Trial	Regimen	No. of patients	ORR	Median PFS (months)	Median OS(months)
CBCSG006 (20)	Gemcitabine + cisplatin	118	64%	7.73	19.37
	Gemcitabine + paclitaxel	118	49%	6.07	18.07
Impassion 130 (18)	Nab-paclitaxel + atezolizumab	451	56.0%	7.2	21
	PD-L1 positive	185	58.9%	7.5	25
	Nab-paclitaxel	451	45.9%	5.5	17
Keynote 355 (19)	Chemotherapy + pembrolizumab	566	NA	7.5	NR
	Chemotherapy + pembrolizumab CPS>10%	136	NA	9.7	NR
	Chemotherapy	211	NA	5.6	NR
E2100 (28)	Paclitaxel + bevacizumab	233	NA	8.8	NA

NA, Not Available; NR, Not Reached.

In a phase 1 study of pucotenlimab (24), PD-L1 expression was detected in 10 tumor samples. As a result, none of the 10 samples were PD-L1 positive. The ORR was 30.0% among these 10 patients, indicating a potential effect of this novel PD-1 antibody in PD-L1-negative patients, although it could not determine whether PD-L1 expression could be a good biomarker for immunotherapy owing to the limited sample size. Future exploratory analyses and large-scale tests may provide us with potential biomarkers for selecting appropriate candidates for immunotherapy.

The ORR in this study was 80.6%, which was much higher than those in both CBCSG 006 and the IMPASSION 130 study. However, the OS has not yet been reached yet at the data cutoff (4 August 2021). Future follow-up and a larger sample size of phase 2 or 3 randomized, controlled studies are needed to identify if improved ORR and PFS could convert to OS benefit in the first-line treatment in mTNBC.

Studies on predictive biomarkers for selecting potential subgroups of immunotherapy are ongoing. PD-L1 expression level, high tumor mutation burden (TMB), and MSI-H have been reported to be potential candidates to predict the efficacy of PD-1/PD-L1 antibodies (29, 30). However, no established predictive biomarkers have been widely accepted in mTNBC to date. Exploratory analysis of tumor samples from this study is in progress.

In conclusion, based on the standard treatment of the GP regimen, pucotenlimab in combination with the GP regimen demonstrated a manageable safety profile with promising anti-tumor activity as first-line treatment in Chinese patients with mTNBC. No unexpected safety signals are observed. Future studies could include the exploration of predictive markers for the addition of pucotenlimab to standard chemotherapy in the mTNBC population.

## Data availability statement

The original contributions presented in the study are included in the article/supplementary material. Further inquiries can be directed to the corresponding author.

## Ethics statement

The studies involving human participants were reviewed and approved by the Ethics Committee of Fudan University Shanghai Cancer Center. The patients/participants provided their written informed consent to participate in this study.

## Author contributions

Study design: JC, XH, JZ, and BW. Drafting of the manuscript: JC and XH. Statistical analysis: JC, XH, and ZT. All authors listed have made a substantial, direct, and intellectual contribution to the work and approved it for publication.

## Funding

This study received funding from the Taizhou Hanzhong Biomedical Co. Ltd. The funder was not involved in the study design, collection, analysis, interpretation of data, the writing of this article or the decision to submit it for publication.

## Acknowledgments

The authors sincerely thank all of the patients, families, nurses, and staff members who participated in our study.

## Conflict of interest

The authors declare that the research was conducted in the absence of any commercial or financial relationships that could be construed as a potential conflict of interest.

## Publisher’s note

All claims expressed in this article are solely those of the authors and do not necessarily represent those of their affiliated organizations, or those of the publisher, the editors and the reviewers. Any product that may be evaluated in this article, or claim that may be made by its manufacturer, is not guaranteed or endorsed by the publisher.
